# Supplementary material for: Diurnal switches in diazotrophic lifestyle increase nitrogen contribution to cereals
Source: Nat Commun. 2023 Nov 18;14:7516. doi: 10.1038/s41467-023-43370-4 (PMC10657418; doi:10.1038/s41467-023-43370-4)
Supplement: Supplementary file 1 — Supplementary Information [file 41467_2023_43370_MOESM1_ESM.pdf]

Diurnal switches in diazotrophic lifestyle increase nitrogen contribution to cereals

Tang *et al.*

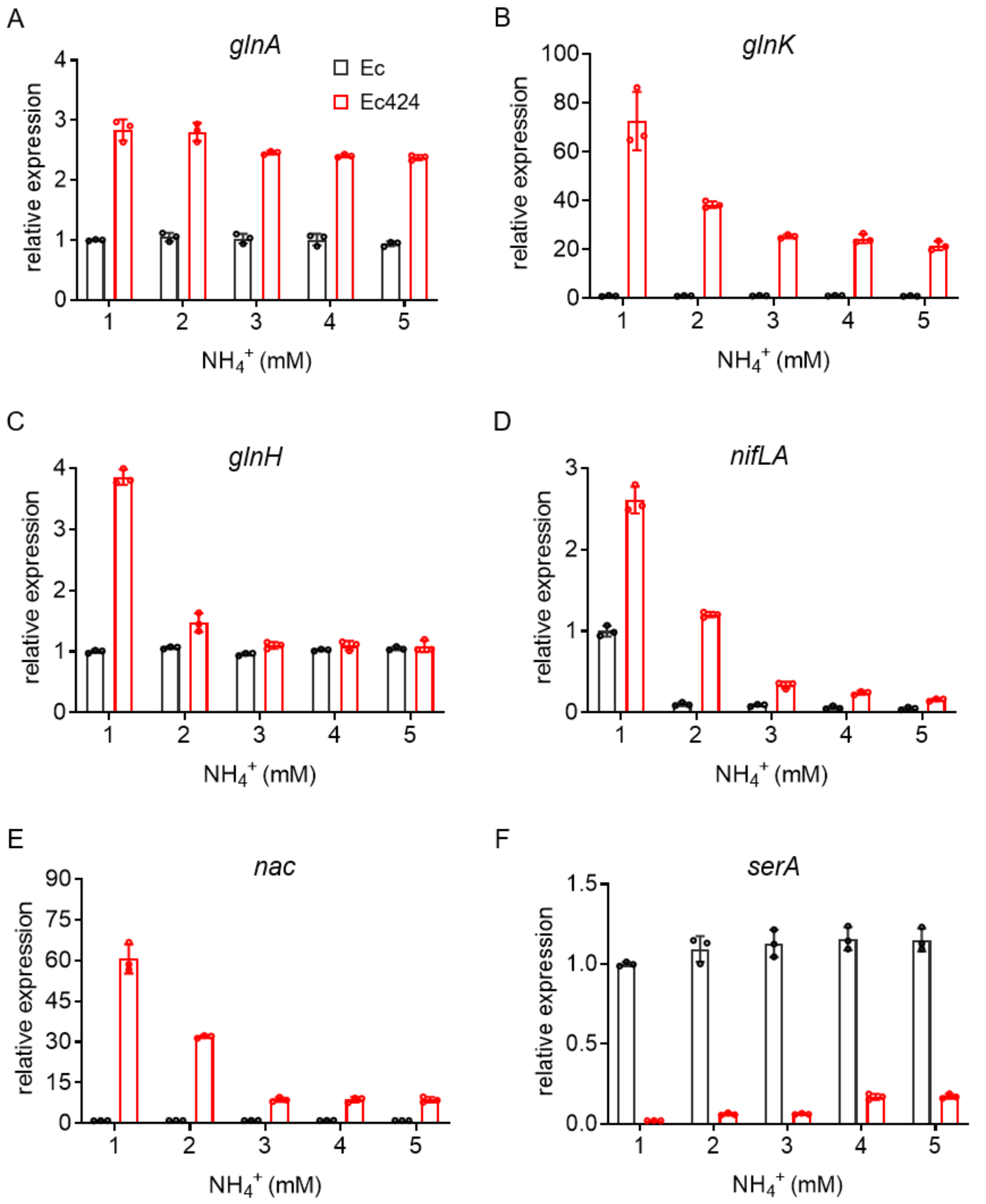

Supplementary Figure 1. Effect of GS-P95L on expression of the NtrC regulated genes indicated above each panel. Bar graphs show the relative quantification of transcripts in the wild type strain Ec (dark gray bars) and the *glnA* variant (GS-P95L) strain Ec424 (red bars). Data are normalized to transcript levels in the Ec strain grown with 1 mM NH_4^+, with a unit reference of 1.0 for each gene. Total RNA was extracted, and equal amounts of RNA were used for absolute quantification by qRT-PCR (in panels **A**, **B**, **C**, **E** and **F**). In panel **D**, *nifLA* expression was measured by *β*-galactosidase assays in strains carrying the P*nifLA::lacZYA* fusion plasmid pKU805. Experiments were carried out in L medium at 30°C with strains grown anaerobically in the presence of the ammonia concentrations indicated on the y axes. The means and SDs were calculated based on at least three biological replicates. Source data are provided as a Source Data file.

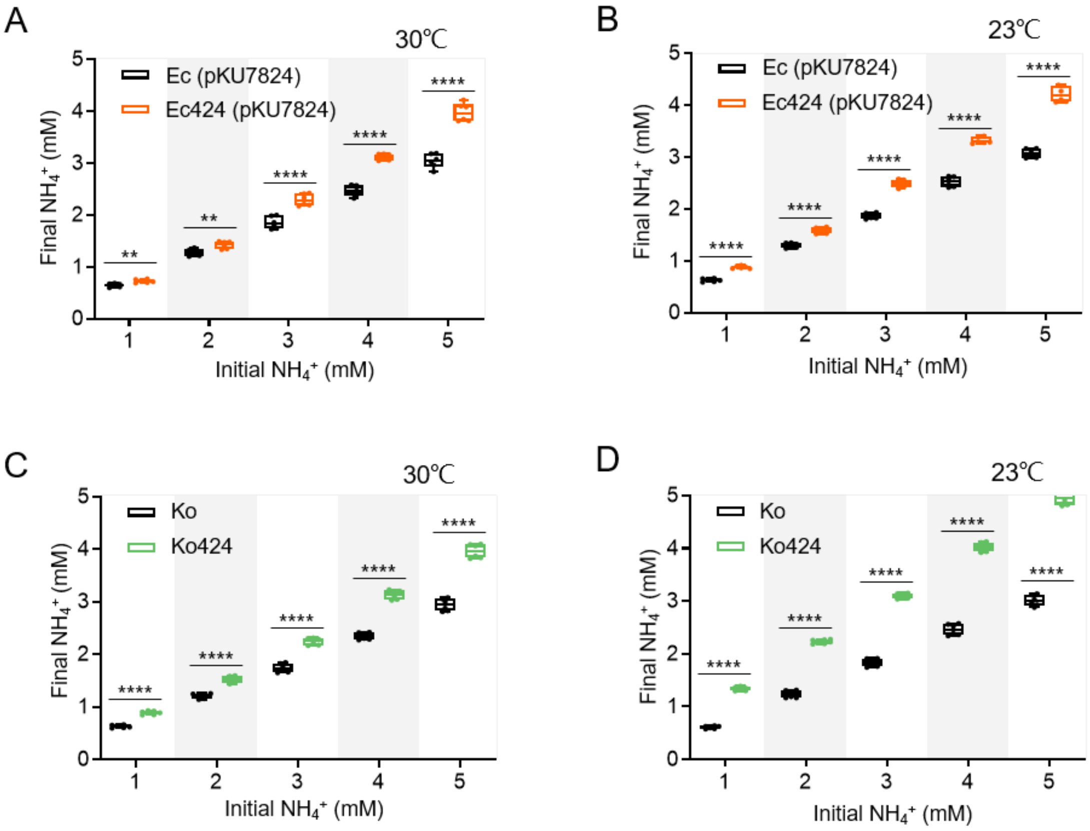

Supplementary Figure 2. Ammonia utilization by strains in relation to temperature. The graphs plot the initial concentration of ammonia in the growth medium (in the range of 1 to 5 mM, x axis) against the final extracellular ammonia concentration (y axis). In all cases the growth conditions correspond to those indicated for the nitrogenase assays in Fig.3 b,c and Fig.4 b,c where ammonium insensitive nitrogen fixation occurs in variant strains encoding the GS-P95L substitution. (**A** and **B**) Comparison of Ec (pKU7824) and Ec424 (pKU7824) at 30°C and 23°C respectively. (**C** and **D**) Comparison of Ko and Ko424 at 30°C and 23°C respectively. The means and SDs were calculated based on at least three biological replicates. Statistical significance is indicated as $^{**}P \leq 0.01$, $^{****}P \leq 0.0001$, analyzed using one-way ANOVA with Student's *t*-test. Source data are provided as a Source Data file.

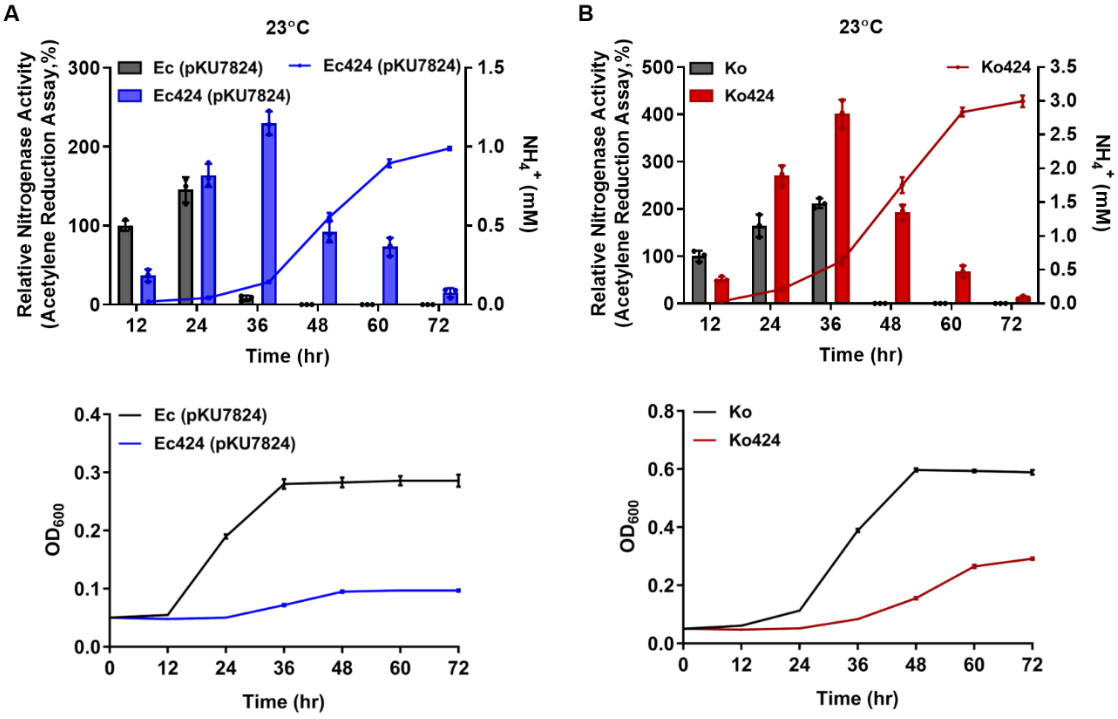

Supplementary Figure 3. Time course of ammonium excretion in comparison with peak nitrogenase activities and growth rates. Strains were grown anaerobically in L medium with glucose as the sole carbon source at 23°C. (**A**) Relative nitrogenase activities of Ec (pKU7824) (dark grey bars) and Ec424 (pKU7824) (blue bars) as indicated on the left y axis. Ammonium in the culture supernatant of Ec424 (pKU7824) is plotted on the right y axis as blue circular symbols connected by a blue line. Ammonia was undetectable from the culture supernatant of wild type Ec (pKU7824). The corresponding growth curves (OD_{600}) of Ec (pKU7824) (black broken line) and Ec424 (pKU7824) (blue line) are shown in the graph below. (**B**) Relative nitrogenase activities of Ko (dark grey bars) and Ko424 (red bars) are plotted on the left y axis. Ammonia in the culture supernatant of Ko424 is plotted on the right y axis (red circular symbols connected by the red line). The corresponding growth curves (OD_{600}) of Ko (black line) and Ko424 (red line) are shown in the graph below. Ammonia was undetectable from the culture supernatant of the wild type Ko strain. Nitrogenase activities were normalized to 100% of the activity exhibited after 12 hours in panels A and B. Means and SDs were calculated based on at least three biological replicates. Source data are provided as a Source Data file.

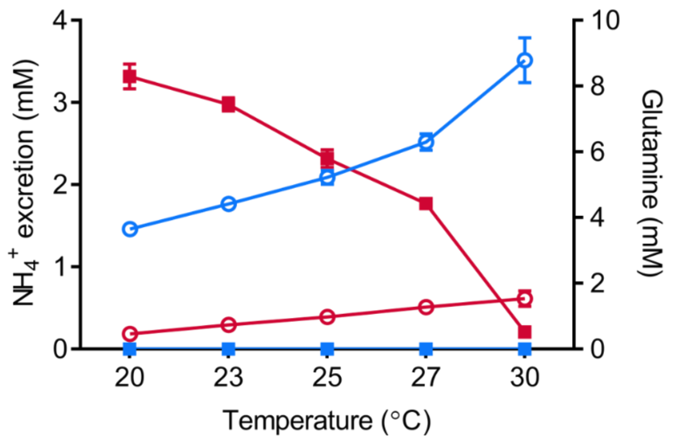

Supplementary Figure 4. Influence of temperature on the internal concentration of glutamine in relation to maximum levels of ammonia excretion in wild type Ko and the Ko424 variant. Intracellular concentrations of glutamine in Ko (open blue circles) and Ko424 (open red circles) are plotted on the right *y* axis respectively. Maximum levels of ammonia excretion in wild type Ko (solid blue squares) and the Ko424 variant (solid red squares) are plotted on the left *y* axis respectively. The means and SDs were calculated based on at least three biological replicates. Source data are provided as a Source Data file.

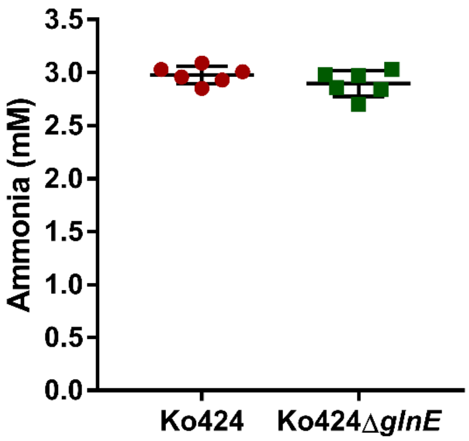

Supplementary Figure 5. Maximum ammonia excretion levels of the Ko424 and Ko424Δ*glnE* strains grown anaerobically in N-free L medium at 23°C. The *glnE* deletion does not influence the concentration of ammonia excreted by Ko424 at 23°C. Means and SDs were calculated based on six biological replicates. Source data are provided as a Source Data file.

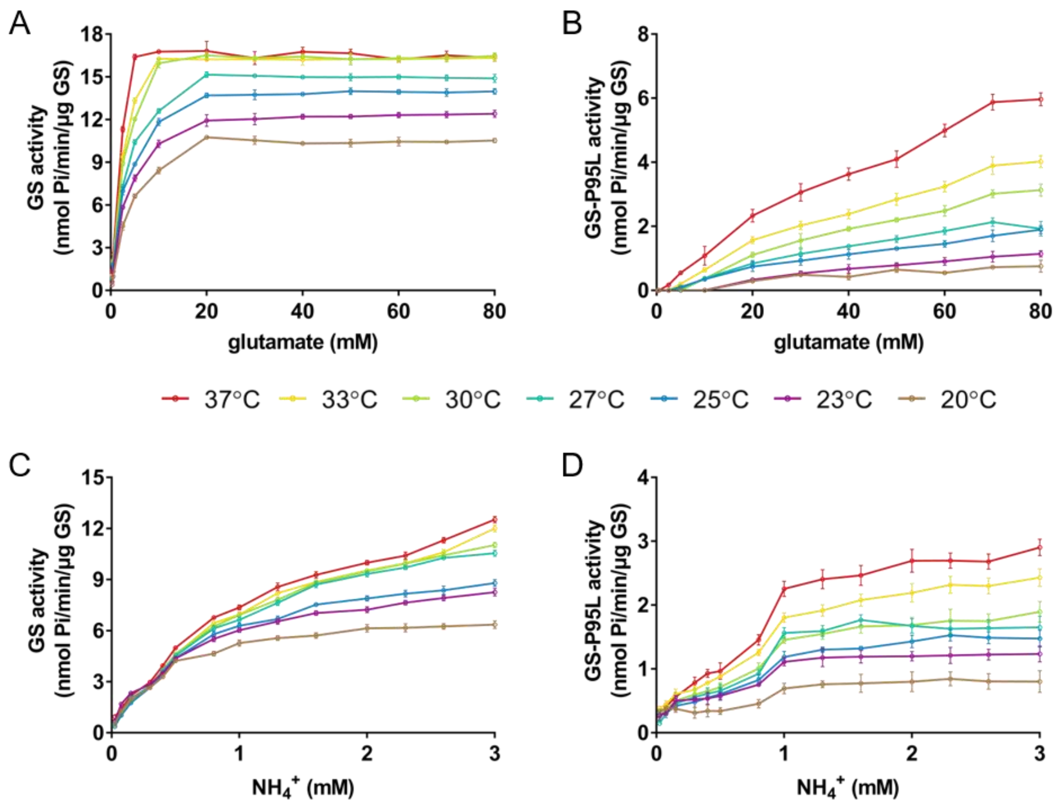

Supplementary Figure 6. ***In vitro*** **catalytic activities of** ***K. oxytoca*** **wild type GS and its variant GS-P95L.** The respective temperatures are color coded as indicated in the graphs and listed in the table in panel E. (**A**), Temperature profile of the wild type GS enzyme from *K. oxytoca* (KoGS) in response to the glutamate concentration. (**B)** Temperature profile of the GS-P95L variant enzyme from *K. oxytoca* (KoGS-424) in response to the glutamate concentration. **(C)** Temperature profile of the wild type GS enzyme in response to the ammonia concentration. (**D)** Temperature profile of the GS-P95L variant enzyme in response to the ammonia concentration. Source data are provided as a Source Data file.

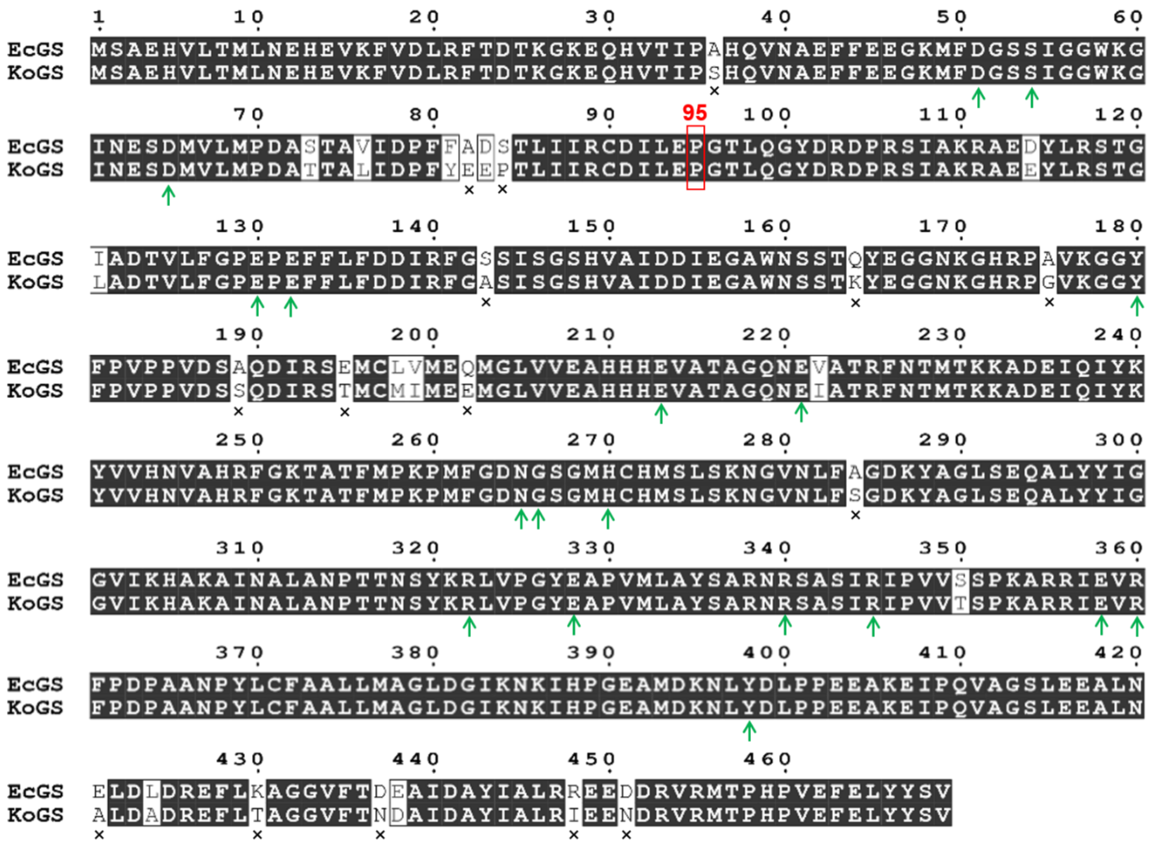

Supplementary Figure 7. Amino acid sequence alignment of glutamine synthetase from *E. coli* (EcGS) and *K. oxytoca* (KoGS). The sequences are 94.2% identical and have 96.8% similarity. Identical amino acids are indicated as solid black boxes, similar amino acids are indicated in hollow black boxes and weakly similar amino acids are indicated by cross. Residues indicated by the green arrow are the active sites of GS involved in catalysis[1,2].

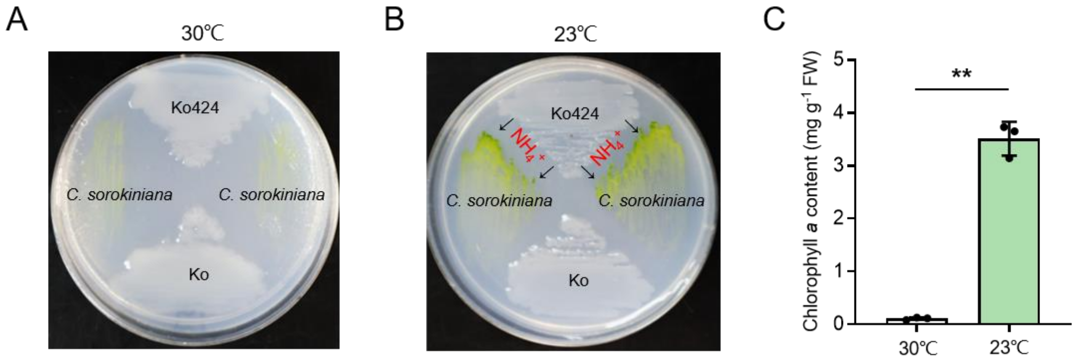

Supplementary Figure 8. Analysis of Ko424 as ammonia donor for growth of *Chlorella sorokiniana*. (**A** and **B**) Growth promotion of the eukaryotic algae *Chlorella sorokiniana* by Ko424 in N-free L agar at 30°C and 23°C, respectively. Strains were streaked in a fan-like arrangement on the plates with *C. sorokiniana* alternating between Ko424 (top) and Ko (bottom) as indicated. Plates were incubated in transparent anaerobic tanks and grown under light illumination for 7 days. (**C**) Chlorophyll *a* content of *C. sorokiniana* adjacent to Ko424 at 30°C and 23°C. Strains were streaked in a fan-like arrangement on the plates with *C. sorokiniana* alternating between Ko424 as in panels **A** and **B**. FW, algae fresh weight. The means and SDs were calculated based on at least three biological replicates. Statistical significance is indicated as $^{**}P \leq 0.01$ analyzed using one-way ANOVA with Student's *t*-test. Source data are provided as a Source Data file.

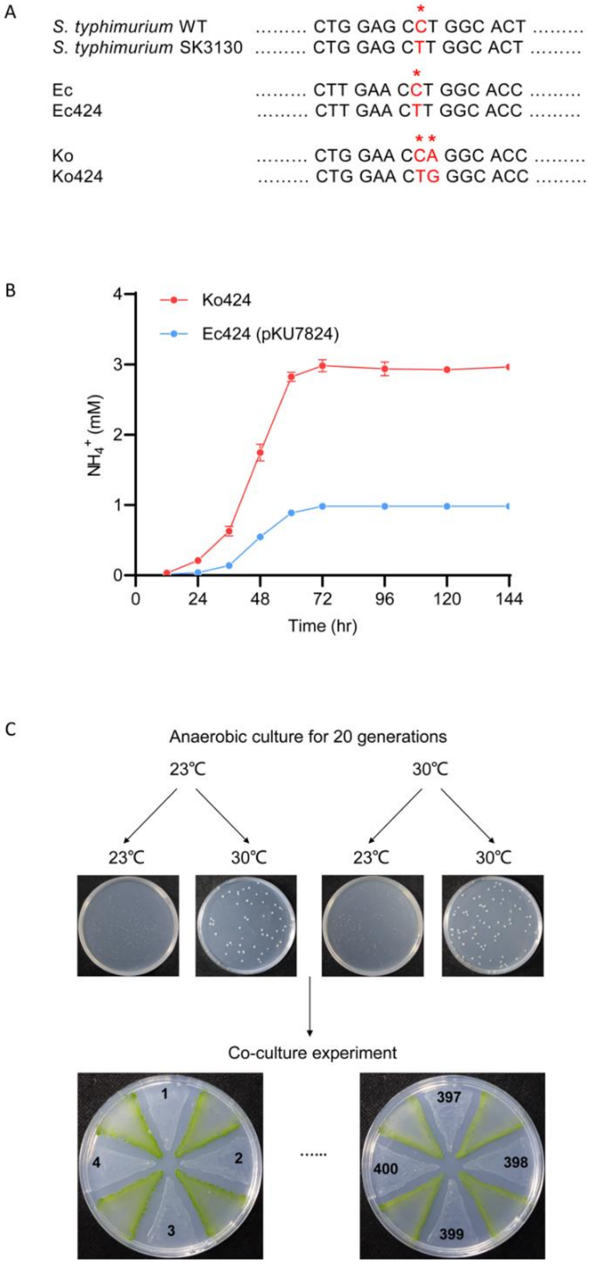

Supplementary Figure 9. Stability of the ammonium excretion phenotype in *K. oxytoca* strain 424. (**A**) Nucleotide substitutions in *S. typhimurium* SK3130, *E. coli* 424 and *K. oxytoca* encoding the GS-P95L variant. **(B)** Ammonia accumulation in the culture supernatant of Ko424 (red) and Ec424 (pKU7824) (blue) during prolonged incubation at 23°C. The means and SDs were calculated based on at least three biological replicates. As many of the data points overlap, standard deviations are not always visible. (**C**) Colony homogeneity (upper figure) and robustness of the ammonia excretion phenotype (lower figure) of Ko424 with the double nucleotide mismatch. Growth conditions and details of the co-culture screening with *Chlorella* in which a total of 400 clones were tested for their ammonia excretion phenotype are listed in Supplementary Method 4. The images shown here are representative of the 20 generations experiment, identical results were obtained after 40 generations. Colonies of Ko424 grown at 23°C are small due to growth inhibition. Source data are provided as a Source Data file.

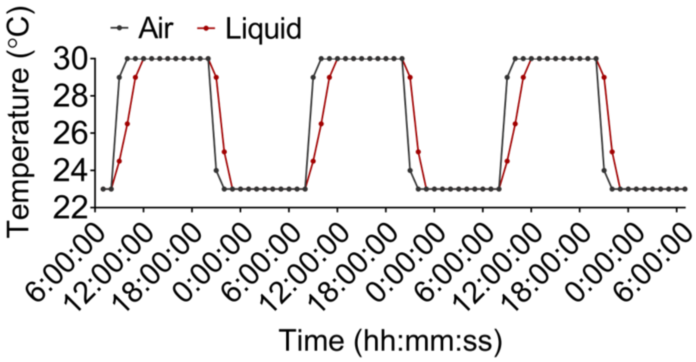

Supplementary Figure 10. Day-and-night temperature changes of the greenhouse chamber air and the hydroponic system fluid. Source data are provided as a Source Data file.

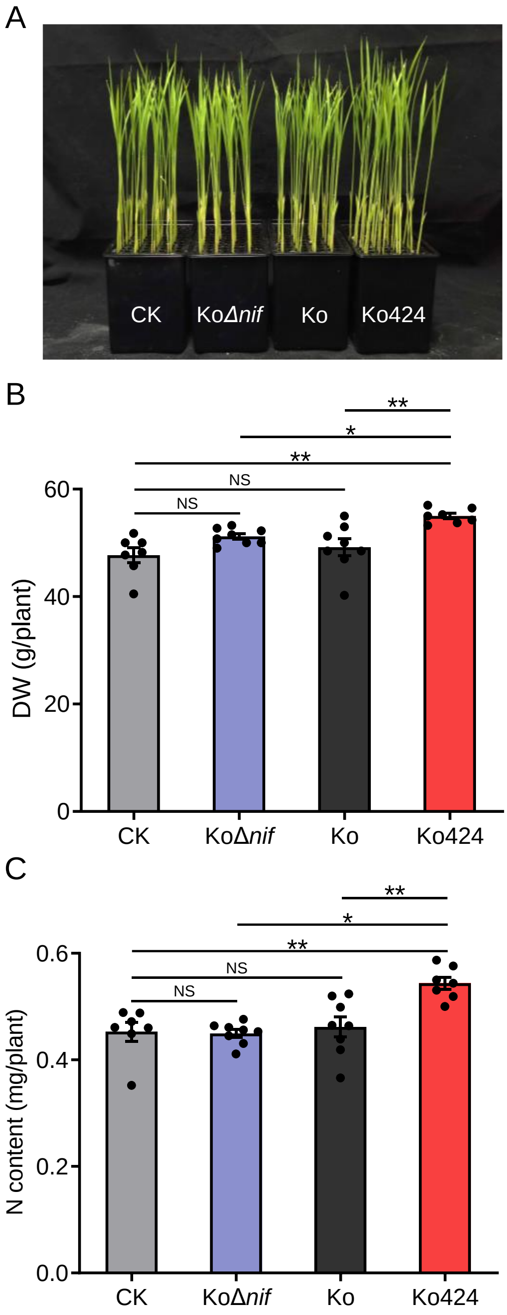

Supplementary Figure 11. Nitrogen contribution of Ko424 to rice grown with 12 hr temperature shifts between day (30°C) and night (23°C). (**A**) Growth phenotype of rice in the absence of a nitrogen source after 12 days inoculation with wild type or mutant strains of *K. oxytoca*. CK indicates no bacteria were added; KoΔ*nif*, inoculation with the *nif* gene cluster deletion mutant of *K. oxytoca*; Ko, inoculation with the wild type strain of *K. oxytoca*; Ko424, inoculation with the GS-P95L mutant strain of *K. oxytoca*. (**B** and **C**) Dry weight and nitrogen content, respectively, of rice after 12 days inoculation with the wild type and mutant strains of *K. oxytoca* indicated as in panel **A.** The means and SEMs were calculated based on at least six biological replicates. Each replicate contained 4 plants. Statistical significance is indicated as $*P \leq 0.05$, $**P \leq 0.01$ analyzed using one-way ANOVA with Student's *t*-test. NS, non-significant. Source data are provided as a Source Data file.

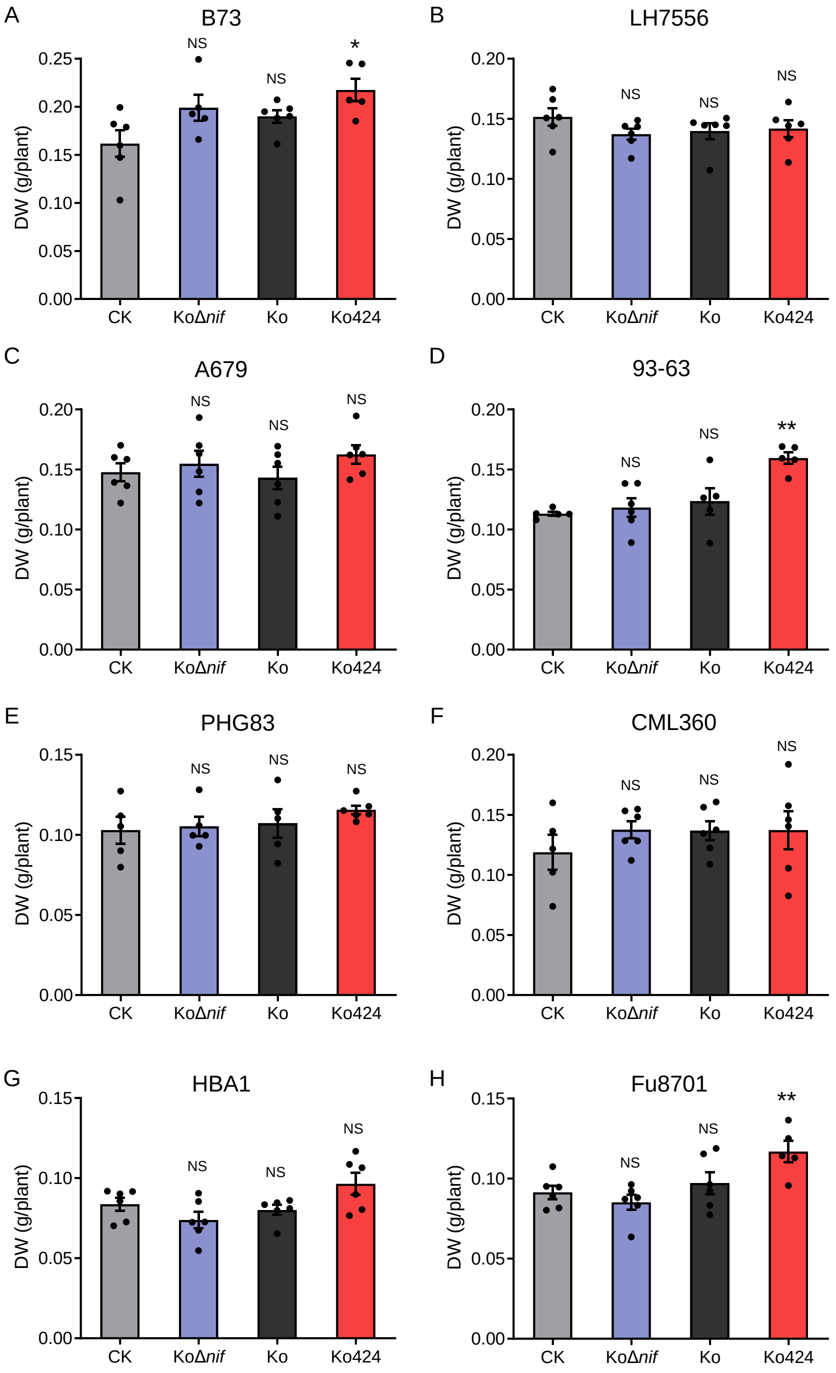

Supplementary Figure 12. Dry weight of 8 different maize inbred lines after inoculation with *K. oxytoca* with temperature shifts between day (30°C) and night (23°C). 8 maize inbred lines (**A**, B73; **B**, LH7556; **C**, A679; **D**, 93-63; **E**, PHG83; **F**, CML360; **G**, HBA1; **H**, Fu8701) were inoculated with *K. oxytoca* for 9 days. CK indicates no bacteria were added; KoΔ*nif*, inoculation with the *nif* gene cluster deletion mutant of *K. oxytoca*; Ko, inoculation with the wild type strain of *K. oxytoca*; Ko424, inoculation with the GS-P95L mutant strain of *K. oxytoca*. The means and SEMs were calculated based on at least five biological replicates. Asterisks indicate a statistically significant difference relative to CK. Statistical significance is indicated as $*P \leq 0.05$, $**P \leq 0.01$ analyzed using one-way ANOVA with Student's *t*-test. NS, non-significant. Source data are provided as a Source Data file.

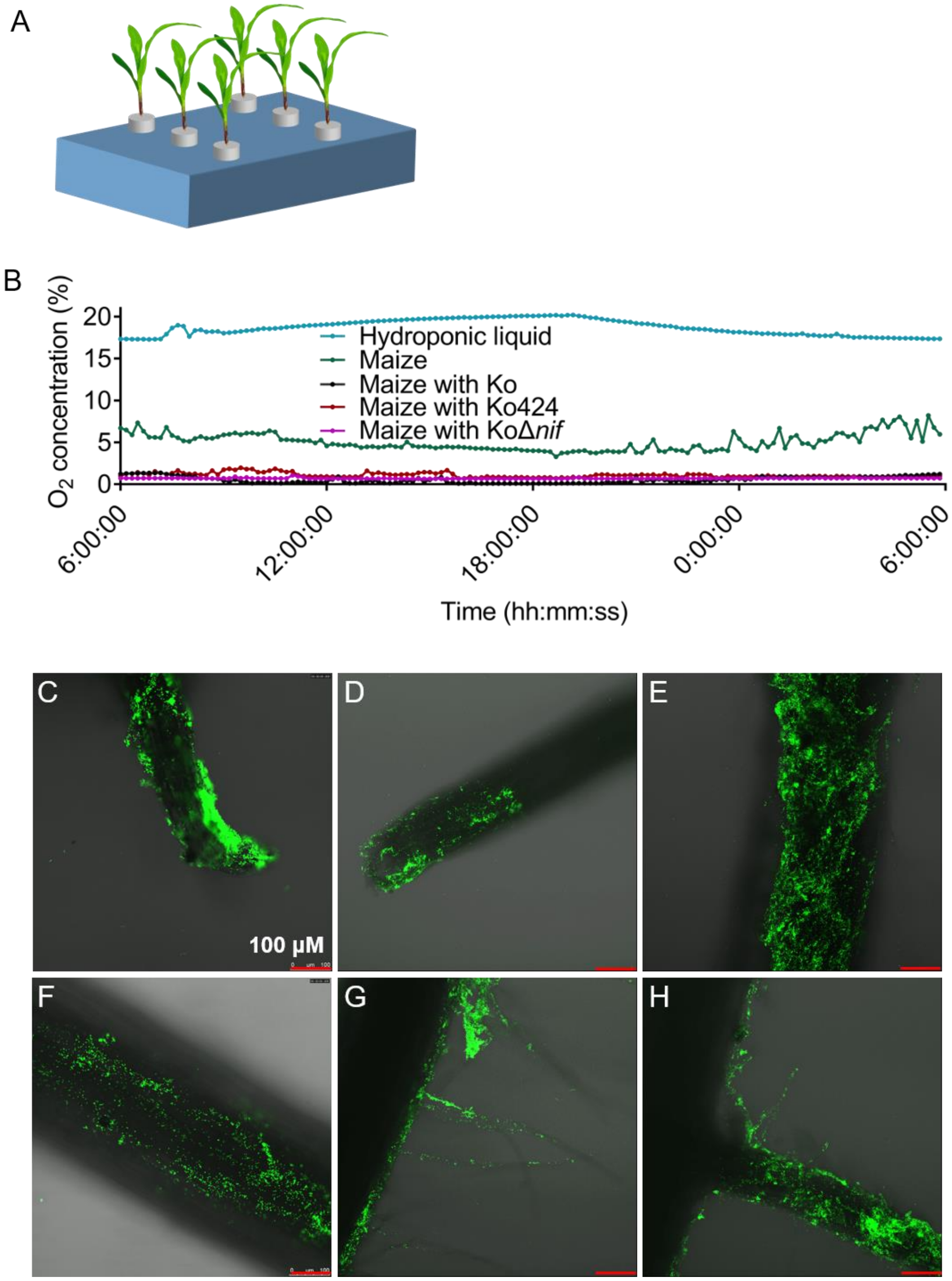

Supplementary Figure 13. Colonization of *K. oxytoca* on the maize 93-63 plant root surface. (**A**) Schematic diagram of the maize hydroponics system (for details see Material and Methods). (**B**) Influence of maize and bacteria (as indicated) on day and night variation of the oxygen concentration in the hydroponic system. (**C**–**H**) Confocal laser scanning micrographs of GFP-expressing cells of wild-type *K. oxytoca* colonized on the maize root surface. C, Root tip of primary root; D, Root tip of lateral root; E, Elongation zone of primary root; F, Elongation zone of lateral root; G, Root hairs; H, Lateral root. Bars = 100 μM. Source data are provided as a Source Data file.

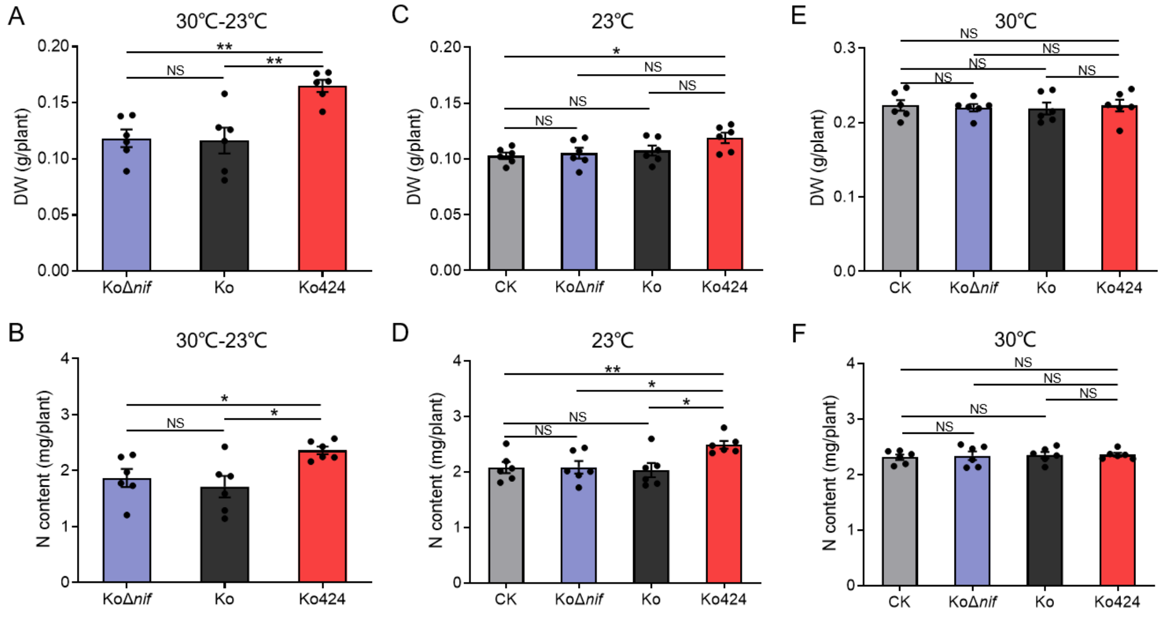

Supplementary Figure 14. Dry weight and nitrogen content of maize after 9 days inoculation with wild type and mutant strains of *K. oxytoca*. Plants were exposed to 12 hr temperature shifts between day (30°C) and night (23°C) (30°C-23°C) (**A** and **B**) or at constant temperature of 23°C (**C** and **D**) or at constant temperature of 30°C (**E** and **F**). CK indicates the uninoculated control. The means and SEMs were calculated based on at least six biological replicates. Statistical significance is indicated as $*P \leq 0.05$, $**P \leq 0.01$ analyzed using one-way ANOVA with Student's *t*-test. NS, non-significant. This is an independent iteration of the experiment reported in the main text in Fig 6 panels b–g. Source data are provided as a Source Data file.

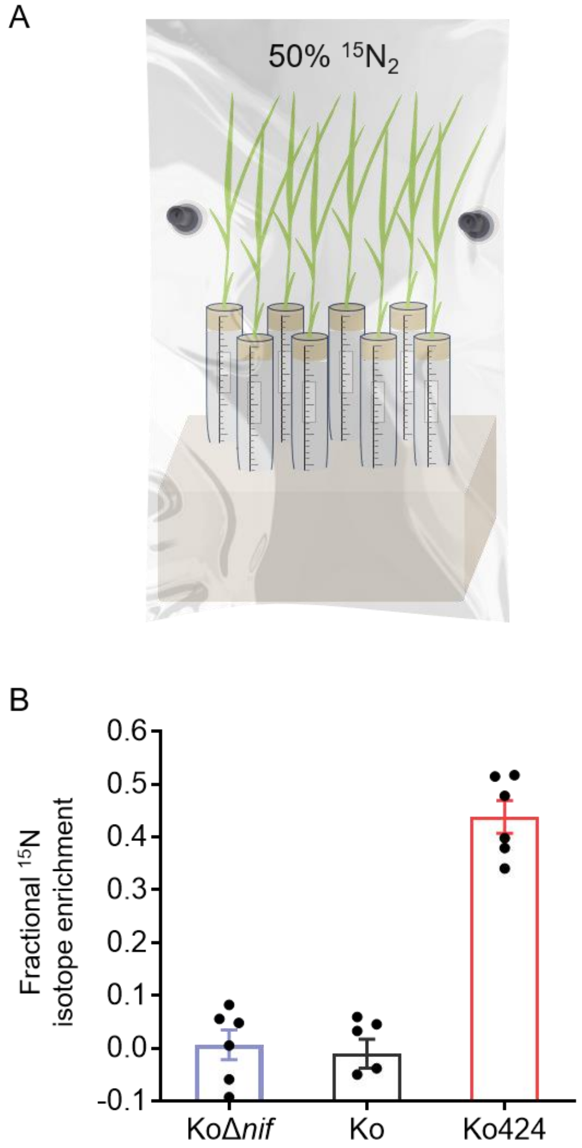

Supplementary Figure 15. ^{15}N isotopic analysis of rice. (**A**) Schematic diagram of the rice hydroponics system for ^{15}N isotopic analysis. (**B**) Determination of the fractional enrichment of the ^{15}N isotope into pheophytin from rice inoculated with KoΔ*nif*, Ko and Ko424. 50% of the co-culture gas was displaced with $^{15}N_2$ gas. 1% CO_2 was added daily. The results were all subtracted from the fractional ^{15}N isotope enrichment of the uninoculated control. KoΔ*nif*, inoculation with the *nif* gene cluster deletion mutant of *K. oxytoca*; Ko, inoculation with the wild type strain of *K. oxytoca*; Ko424, inoculation with the GS-P95L mutant strain of *K. oxytoca*. The means and SEMs were calculated based on at least six biological replicates. Asterisks indicate a statistically significant difference relative to Ko. Statistical significance is indicated as $^{**}P \leq 0.01$ analyzed using one-way ANOVA with Student's *t*-test. Source data are provided as a Source Data file.

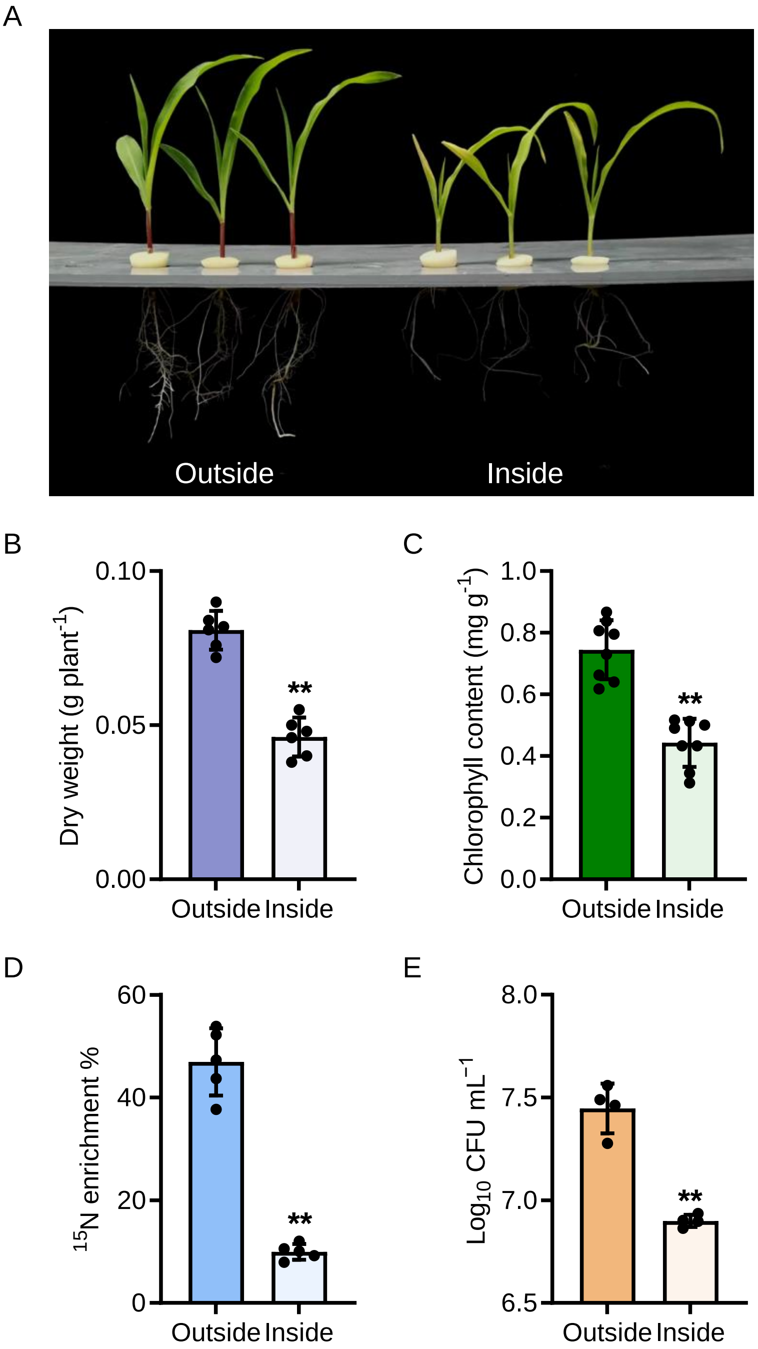

Supplementary Figure 16. Physiological differences between maize grown outside and inside the gas-tight bag after 6 days. (**A**) Influence of growth outside and inside the gas-tight bag on the growth phenotype of the maize inbred line 93-63 after 6 days. 0.5 mM $^{15}NO_3^-$ was added after germination. (**B–D**) Dry weight, chlorophyll content and $^{15}NO_3^-$ absorption of maize grown outside and inside the gas-tight bag. (**E**) *K. oxytoca* proliferation in Hoagland nutrient solution containing the corresponding root exudates (used as sole carbon source) from maize grown outside and inside the gas-tight bag. The number of *K. oxytoca* cells (CFU) relate to 24 hours of growth. The means and SDs were calculated based on at least five biological replicates. Statistical significance is indicated as $^{**}P \leq 0.01$ analyzed using one-way ANOVA with Student's *t*-test. Source data are provided as a Source Data file.

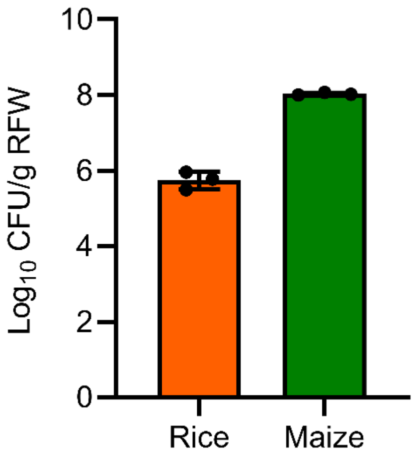

Supplementary Figure 17. Ko424 proliferation in the Kimura B nutrient solution (rice), or Hoagland nutrient solution (maize), containing the corresponding root exudates from rice or maize. The number of Ko424 cells (CFU) relate to 24 hours of growth. CFU, colony forming unit; RFW, root fresh weight. The means and SEMs were calculated based on at least three biological replicates. Source data are provided as a Source Data file.

Supplementary Table 1. Properties of the GS -P95L substitution in *E. coli*. Growth rate, intracellular glutamine and glutamate levels and *glnA* gene expression levels of wild type *E. coli* (Ec) and the GS-P95L point mutant of *E. coli* (Ec424) grown with 15 mM NH_4^+ under the conditions indicated in the Table. *β*-galactosidase activities (*β*-Gal) were obtained with the indicated *E. coli* strains carrying the plasmid P*glnA::lacZYA*. Means and SDs were calculated based on at least three biological replicates. Source data are provided as a Source Data file.

**Strain**	**Doubling time (min)**	**Glutamine (mM)**	**Glutamate (mM)**	***β*-Gal (Miller units; P*glnA-lacZYA*)**
Aerobic, 37°C				
Ec	57±0.5	4.9±0.5	52±3	100% (1463±37)
Ec424	66±0.3	1.8±0.3	53±4	235% (3448±51)
Anaerobic, 30°C				
Ec	138±5.1	4.4±0.2	46±3	100% (1266±30)
Ec424	209±9.6	1.0±0.1	41±2	219% (2771±43)

Supplementary Table 2. Influence of temperature on growth rate of strains and maximum ammonia excretion levels in *E. coli* containing the *K. oxytoca* engineered nitrogen fixation gene cluster on plasmid pKU7824 anaerobically in L medium. N.D indicates not detectable. Note that the ammonia excretion values do not correspond to the final ammonium concentrations in Supplementary Fig. 2A and B, since maximum ammonium excretion is observed after a much longer incubation period. Source data are provided as a Source Data file.

	**Doubling time (min)**		**Maximum NH_4^+ excretion (mM)**	
	**Ec (pKU7824)**	**Ec424 (pKU7824)**	**Ec (pKU7824)**	**Ec424 (pKU7824)**
33°C	433±15	812±17	N.D.	N.D.
30°C	245±17	677±11	N.D.	N.D.
27°C	286±11	724±13	N.D.	0.51±0.04
25°C	349±16	790±25	N.D.	0.70±0.05
23°C	412±15	875±42	N.D.	1.02±0.05
20°C	495±27	1081±39	N.D.	1.13±0.07

Supplementary Table 3. Influence of temperature on growth rates when strains are cultured aerobically in L medium containing 15 mM NH_4^+. Source data are provided as a Source Data file.

	Doubling time (min)		Doubling time (min)	
	**Ec (pKU7824)**	**Ec424 (pKU7824)**	**Ko**	**Ko424**
37°C	61±1	84±1	53±2	87±4
33°C	72±1	92±1	55±2	91±4
30°C	86±1	116±1	59±2	95±4
27°C	105±1	142±1	73±3	113±4
25°C	126±1	177±1	94±2	182±4
23°C	144±1	197±1	104±5	393±19
20°C	207±3	299±3	162±5	959±38

Supplementary Table 4. Affinity constants calculated from the catalytic activity of the wild type GS from *K. oxytoca* for the substrates glutamate (Glu) and ammonia (NH_4^+) at the indicated temperatures.

Temperature	K_m(Glu) (mM)	K_m(NH_4^+) (mM)
20°C	11.18	0.21
23°C	6.52	0.17
25°C	4.29	0.14
27°C	4.17	0.13
30°C	3.98	0.12
33°C	3.68	0.12
37°C	3.53	0.11

Supplementary Table 5. Temperature profile of Zhengzhou, Henan, China.

Location	Month	Annual mean daily max temp. (°C)	Annual mean daily min temp. (°C)
Zhengzhou, Henan	June	31.8	20.4
	July	31.8	23.1
	August	30.5	22.2

Supplementary Table 6. Bacterial strains used in this study.

Strains	Relevant characteristics	Source
SK3130	*Salmonella typhimurium*, glutamine auxotroph	Ikeda *et al.* [3]
Ec	*Escherichia coli* NCM3722, wild type	This lab
Ec_rif^R	Rifampicin resistant *Escherichia coli* NCM372	This work
Ec424	Ec derivative, *glnA* P95L mutant	This work
Ec (pKU7824)	Ec derivative carrying the entire *nif* gene cluster of Ko	This work
Ec424 (pKU7824)	Ec424 derivative carrying the entire *nif* gene cluster of Ko	This work
Ko	*Klebsiella oxytoca* M5al, wild type	This lab
Ko424	Ko derivative, *glnA* P95L mutant	This work
KoΔ*nif*	Ko derivative, *nif* gene cluster deletion mutant	This work
Ko424Δ*glnE*	Ko424 derivative, *glnE* deletion mutant	This work
Top10	F$^-$ *mcr*A Δ(*mrr*-*hsd*RMS-*mcr*BC) φ80 *lac*ZΔM15 Δ*lac*X74 *nup*G *rec*A1 *ara*D139 Δ(*ara*-*leu*)7697 *gal*E15 *gal*K16 *rps*L(Str^R) *end*A1 λ$^-$. Used for construction and propagation of plasmids	This lab
ST18	*E. coli* S17 λ pir Δ*hemA*. Used for conjugations	This lab
BL21(DE3)	*E. coli* F$^-$ *omp*T *hsd*S($r_B^- m_B^-$) *gal dcm*(DE3).	This lab
BL21(DE3)Δ*glnA*Δ*glnE*	BL21(DE3) derivative, *glnA* and *glnE* double-deletion mutant. Used for protein expression	This work

Supplementary Table 7. Nucleotide sequence of qRT-PCR primers.

ID	Sequence 5' → 3'	Application
Ec16S-F	AGAATGCCACGGTGAATACG	RT-qPCR *E. coli* 16S rRNA
Ec16S-R	CTACGGTTACCTTGTTACGACTTC	
EcglnA-F	CGTTCTGCGTCTATCCGTATTC	RT-qPCR *E. coli glnA*
EcglnA-R	CCAGTTCGTTCAGTGCTTCTT	
EcglnK-F	AGCGTTATCTTCCATTGGTATTCA	RT-qPCR *E. coli glnK*
EcglnK-R	TCATCAGCAATCGCCACATC	
EcglnH-F	GGCGGGCATTACCATCAC	RT-qPCR *E. coli glnH*
EcglnH-R	CCAGTTCCATATAGGCGTTATCG	
Ecnac-F	GCGTTGATGTGAATGCTATTGC	RT-qPCR *E. coli nac*
Ecnac-R	AGGAGTGGTAATGCGTGACA	
EcserA-F	GCGAACTGCTGCTGCTAT	RT-qPCR *E. coli serA*
EcserA-R	GGTGGACGGATTCTCTGGTA	

Supplementary Method 1. RNA purification and quantitative RT-PCR

Cells were incubated under anaerobic conditions as described above, approximately 5 × 10^8 cells were harvested during mid-log phase and immediately twice the volume of RNAprotect Bacteria Reagent (QIAGEN; 76506) was added. The mixture was then vortexed for 5 s, incubated for 5 min at room temperature and extracted immediately (or briefly stored at 4°C) prior to RNA extraction using the RNeasy Midi Kit (QIAGEN; 74104). Genomic DNA in the RNA solution extracted in the previous step was removed using TURBO DNA-*free*™ DNAse (Ambion; AM1907) following rigorous DNase treatment according to the manufacturer's instructions. cDNA synthesis was performed with High-Capacity RNA-to-cDNA™ Kit (Thermo Fisher Scientific; 4387406) using up to 2 μg of total RNA per 20 μL reaction as recommended by the manufacturer. The resulting 1 μL cDNA was used as template in a 20 μL qPCR reaction performed with the PowerUp™ SYBR™ Green Master Mix (Thermo Fisher Scientific; A25742) and the real-time quantitative PCR instrument (Thermo Fisher Scientific, Applied Biosystems™ QuantStudio™ 5 Real-Time PCR System). Relative quantification of target genes (*glnA*, *glnK*, *glnH*, *nifLA*, *nac* and *serA*) alongside the reference house-keeping gene (16S rRNA) was performed by the $2^{\Delta Cq \Delta Cq}$ method[4]. The primers used are listed in Supplementary Table 7.

Supplementary Method 2. Protein expression and purification

The *glnA* genes were amplified from the genomes of Ko and Ko424 and inserted into the expression vector, pET-28a, to produce the recombinant plasmids pET-28a-KoGS and pET-28a-Ko424GS. The resulting plasmids were transformed into *E. coli* BL21 (DE3) Δ*glnA*Δ*glnE*, and transformed clones were selected on LB plates supplemented with 50 μg mL^{-1} kanamycin. For purification of His-tagged GS proteins, the fresh transformants were pre-cultivated in LB medium overnight and transferred into 2 L LB medium supplemented with 50 μg mL^{-1} kanamycin in a ratio of 1:100. After incubation at 37°C to an optical density at 600 nm of 0.6, cultures were induced by adding 150 μM IPTG and incubated at 18°C for 16 hours. Then, the induced cells were collected by centrifugation, resuspended in sonication buffer (50 mM Tris-HCl, 200 mM NaCl, 20 mM imidazole, 1 mM dithiothreitol, pH 8.0) and sonicated for 20 min on ice using a sonifier. Cell lysates were clarified via centrifugation at 10,000 rpm for 30 min to remove cell debris and insoluble material and loaded onto a 5 ml HisTrap HP column (GE Healthcare; 17-5248-02). Next, proteins were eluted using a linear gradient of

imidazole (20 to 500 mM) in the same buffer (50 mM Tris-HCl, 200 mM NaCl, pH 8.0) using Amersham Biosciences ÄKTA FPLC System. Eluted fractions were analyzed by 12% SDS-PAGE, and the fractions containing the protein of interest were pooled and dialyzed in storage buffer (50 mM Tris-HCl, pH 8.0). All the proteins were stored in small aliquots supplemented with 10% glycerol at −80°C.

Supplementary Method 3. GS activity assay

Biosynthetic activity of purified GS proteins was determined by measuring the production of inorganic phosphate that resulted from the hydrolysis of ATP[5]. Briefly, each reaction mixture contained 100 mM MOPS (pH 8.2), 10 mM ATP, 50 mM $MgCl_2 \cdot 6H_2O$, 250 mM L-glutamate, 50 mM NH_4Cl and purified unadenylylated GS in a final volume of 100 µL. To determine the substrate affinity of GS, glutamate and ammonia were diluted to appropriate concentrations. The reaction was initiated by adding enzyme solution and stopped following a 1–5 min incubation at different temperatures with 300 µL Solution D, which was a mixture of two parts Solution A (12% L-ascorbic acid in 1N HCl) and one part Solution B (2% ammonium molybdate tetrahydrate), to generate color for 5 min. Then 300 µL of 2% sodium citrate tribasic dihydrate in 2% acetic acid was added to quench further color development. The product of the reactions was measured at 655 nm using a plate reader (BioTek Synergy neo multi-detection microplate reader).

Supplementary Method 4. Stability studies of Ko424

Ko424 was anaerobically cultured in L medium for either 12 days or 24 days at 23°C, and either 6 days or 12 days at 30°C to enable either ~20 or ~40 cell divisions, respectively. During this period, the bacteria were diluted every 3 days in fresh L medium. The cells were then diluted on solid L medium and anaerobically cultured at 23°C and 30°C until the colony size remained unchanged. 100 clones were randomly selected from each set of plates representing the 4 conditions indicated in Supplementary Fig 9, resulting in the screening of a total of 400 clones. Suspensions of the selected 400 clones at similar cell density were streaked in a fan-shaped pattern adjacent to *Chlorella sorokiniana* on solid L medium with glucose as carbon source. The plates were placed in a transparent anaerobic tank and grown in an illuminated incubator either at 23°C for one week. The growth and greening of *Chlorella* adjacent to Ko424 indicated that after either 20 or 40 generations, Ko424 still maintained the

ability to excrete ammonia.

Supplementary Method 5. Rice culture and medium

Rice (*Japonica*) was grown in a hydroponic system. Plants were cultured in the greenhouse with photoperiod of 12 hours between light and dark intervals. The incubation temperature was 30°C in the light (7 am to 7 pm) and 23°C in the dark (7 pm to 7 am) with humidity maintained at ~60–70%. Rice was cultured in Kimura B nutrient solution, which consists of 0.18 mM KH_2PO_4, 0.55 mM KH_2PO_4, 90 μM K_2SO_4, 0.37 mM $CaCl_2·2H_2O$, 50 μM EDTA-Fe(II), 1.6 mM $Na_2SiO_3·9H_2O$, 9.14 μM $MnCl_2·4H_2O$, 46.2 μM H_3BO_3, 0.32 μM $CuSO_4·5H_2O$, 2.73 μM $Na_2MoO_4·2H_2O$, 0.76 μM $ZnSO_4·7H_2O$, 0.76 μM $ZnCl_2$, 0.32 μM $CuSO_4$, and 1‰ MES with the pH adjusted to 6.5. No additional carbon source was added during all cultivations of rice and maize.

Supplementary Method 6. Rice inoculation and growth measurement

Rice seeds were soaked with 2% NaClO and disinfected for 30 minutes, then cleaned repeatedly with sterilized distilled water for 4–5 times to remove disinfectant, germinated at 30°C for 2 days and then grown for 4 days after adding 2 mM KNO_3. After this initial plant growth period, the complete root system of the rice was incubated for 1 hour with a suspension of inoculant bacteria at a bacterial cell density of 10^9 cells per mL. Note: the bacterial cells were prepared as following: the bacteria were grown in LB medium to an OD_{600} of 0.6–0.8 in the log phase. Subsequently, the bacterial cells were harvested by centrifugation, washed and resuspended in corresponding plant nutrient solution to the required cell density (10^9). Plants were then transferred back to the hydroponic system and grown for a further 12 days prior to harvesting. 24 plants were grown per pot which filled with 1 liter nutrient solution. To determine plant dry weight, plants were placed into kraft paper bags, and dried in an oven at 80°C until the weight no longer changes. To measure nitrogen content, plant tissues were ground into a powder with a mortar and pestle, and analyzed for total nitrogen using an elemental analyzer (vario MACRO cube CHNOS Elemental Analyzer, Elementar Analysensysteme GmbH, Hanau, Germany).

Supplementary Method 7. Bacteria growth in root exudates

To prepare root exudates, roots obtained from rice seedlings germinated for 9 days, or maize seedlings germinated for 7 days were immersed in the Kimura B nutrient solution

(rice), or Hoagland nutrient solution (maize) for 24 hours under aseptic conditions. *K. oxytoca* proliferation in nutrient solution containing the corresponding root exudates from rice or maize was determined by inoculating equivalent numbers of bacteria into the exudates with 1 mM ammonia added as nitrogen source and no additional carbon source. After 24 hours cultivation at 30°C with 160 rpm shaking, the number of bacteria were counted as colony forming units.

Supplementary references

1. Eisenberg, D., Gill, H. S., Pfluegl, G. M. & Rotstein, S. H. Structure-function relationships of glutamine synthetases. *Biochim. Biophys. Acta.* **1477**, 122–145 (2000).
2. van Heeswijk, W. C., Westerhoff, H. V. & Boogerd, F. C. Nitrogen assimilation in *Escherichia coli*: putting molecular data into a systems perspective. *Microbiol Mol. Biol. Rev.* **77**, 628–695 (2013).
3. Ikeda, T. P., Shauger, A. E. & Kustu, S. *Salmonella typhimurium* apparently perceives external nitrogen limitation as internal glutamine limitation. *J. Mol. Biol.* **259**, 589–607 (1996).
4. Livak, K. J. & Schmittgen, T. D. Analysis of relative gene expression data using real-time quantitative PCR. *Methods* **25**, 402–408 (2002).
5. Gawronski, J. D. & Benson, D. R. Microtiter assay for glutamine synthetase biosynthetic activity using inorganic phosphate detection. *Anal. Biochem.* **327**, 114–118 (2004).
