# Supplementary material for: Diurnal switches in diazotrophic lifestyle increase nitrogen contribution to cereals
Source: Nat Commun. 2023 Nov 18;14:7516. doi: 10.1038/s41467-023-43370-4 (PMC10657418; doi:10.1038/s41467-023-43370-4)
Supplement: Supplementary file 3 — Description of Additional Supplementary Files [file 41467_2023_43370_MOESM3_ESM.pdf]

Description of Additional Supplementary Files

File Name: Supplementary Data 1
Description: Protein accession numbers of GS from g-proteobacteria for multiple sequence alignment and Weblogo generation in Figure 2b.
